# Effect of Nano-TiO_2_ Dioxide on the Hydration Process of Tunnel Construction in Low-Temperature Environments

**DOI:** 10.3390/nano16020138

**Published:** 2026-01-20

**Authors:** Yongchun Jiao, Huijian Chen, Shengfu Xu, Fei Fu, Yipeng Tao, Sheng’ai Cui

**Affiliations:** 1 Third Engineering Bureau, China Anneng Group, Chengdu 611135, China; z1913959912@126.com (Y.J.); 13722189630@163.com (H.C.); 18982924086@163.com (S.X.); 2 School of Civil Engineering, Southwest Jiaotong University, Chengdu 610032, China; bai13340823900@my.swjtu.edu.cn (F.F.); yipengtao@my.swjtu.edu.cn (Y.T.)

**Keywords:** nano-TiO_2_, low-temperature curing, mechanical properties, hydration process, detailed observation of pore structure, differential thermal analysis

## Abstract

To address winter construction challenges such as slow early-stage strength development, inhibited hydration processes, and pore structure defects in concrete under low-temperature conditions, this study employs nano-TiO_2_ as a modifying agent. It is incorporated into concrete through cement replacement methods; the study systematically investigates the influence of different admixture dosages (1%, 2%, 3%, by cement mass) on the mechanical properties, hydration process, and micro-pore structure of concrete. The test employed an electro-hydraulic servo universal testing machine to measure compressive and splitting tensile strengths. Differential thermal analysis (DTA) characterized the formation of hydration products (Ca(OH)_2_). Micro-CT technology and pore network modeling were utilized to quantify micro-pore parameters. Results indicate that (1) nano-TiO_2_ regulates the setting time of pure paste, with increased dosage shortening both initial and final setting times. At a 3% dosage, initial setting time plummeted from 5.5 min in the control group to 3.3 min; (2) nano-TiO_2_ significantly enhances early-age (1–3 days) strength of low-temperature concrete, with optimal effect at 1% dosage. Compressive strength and splitting tensile strength at 1 day increased significantly by 20% and 26%, respectively, compared to the control group. Strength differences among groups gradually narrowed at 28 days; (3) DTA indicates that nano-TiO_2_ accelerates early cement hydration; (4) micro-CT results show that the 1% dosage group exhibits significantly reduced porosity at day 1 compared to the control group, with notable decreases in Grade 0 and Grade 1 interconnected porosity resulting in the most optimal pore structure density. In summary, the optimal dosage of nano-TiO_2_ in low-temperature environments is 1% by mass of cement. Through the synergistic “nucleation-filling effect,” it promotes early-stage hydration and optimizes pore structure, providing technical support for winter concrete construction.

## 1. Introduction

During tunnel construction in cold regions, the sprayed concrete at the portal section is highly susceptible to the effects of large temperature differentials and severe cold weather [1]. Low temperatures (typically below 5 °C) significantly inhibit the cement hydration process [2], resulting in slow early-stage strength development and prolonged setting times in concrete [3]. Simultaneously, inadequate development of the internal pore structure can lead to reduced water resistance and freeze–thaw resistance [4], posing serious threats to the construction safety and long-term durability of engineering structures. In tunnel construction projects in cold regions, shotcrete must rapidly develop strength at low temperatures to achieve initial support. However, low temperatures prolong the cement hydration induction period by 3 to 5 times [5,6], causing the concrete rebound rate to rise above 25% and resulting in a 28-day strength retention rate below 80% [7,8]. Therefore, how to enhance the early-stage performance of concrete in low-temperature environments through material modification techniques has become a core research focus for addressing construction challenges in cold regions.

To improve the performance of concrete at low temperatures, scholars both domestically and internationally have conducted extensive research, with mineral admixture modification being one of the most commonly employed technical approaches [9,10]. Traditional mineral admixtures (such as silica fume and fly ash) can optimize the late-stage pore structure of concrete through pozzolanic reactions. However, their reactivity is significantly suppressed in low-temperature environments, resulting in limited enhancement of early-stage strength [11,12,13]. In recent years, nanomaterials have demonstrated unique advantages in the modification of cement-based materials due to their “nano effects”: high specific surface area and high surface activity [14,15,16,17]. For example, nano-SiO_2_ can accelerate cement hydration through nucleation effects [18,19]. At a 2% dosage in a 5 °C environment, it increases concrete’s 1-day compressive strength by 39%. However, a high dosage of nano-TiO_2_ may accelerate the setting rate of the cement matrix, thereby impeding its practical application [20,21,22,23]. Nano-CaCO_3_ can refine pores through a filling effect, but its regulatory effect on cement hydration at low temperatures is weak, with early strength gains below 15% [24,25,26]. In contrast, nano-TiO_2_ not only exhibits surface activity comparable to nano-SiO_2_, but its anatase structure also maintains high chemical stability at low temperatures [27]. Furthermore, studies indicate that nano-TiO_2_ can enhance the anti-fouling properties of concrete at ambient temperatures through photocatalytic effects [28], thereby improving long-term durability and expanding the functional boundaries of concrete [29,30].

Therefore, nano-TiO_2_ also holds significant research value in modifying mineral admixtures. Li et al. [31] investigated the effects of nano-TiO_2_ on the mechanical properties and microstructure of ultra-high performance concrete. The results indicate that the nucleation effect and filling effect of nano-TiO_2_ can enhance the strength of ultra-high performance concrete. Simultaneously, nano-TiO_2_ reduces the orientation of Ca(OH)_2_, resulting in denser concrete with decreased porosity and micro-cracks. Chen et al. [32] studied the effects of incorporating two different types of nano-TiO_2_ on the properties of cement paste and mortar. Research indicates that during the hydration process, although nano-TiO_2_ does not participate in the hydration reaction itself, it acts as a catalyst to accelerate the hydration of cementitious materials. Simultaneously, it reduces the overall porosity of the mortar, alters the pore size distribution, and enhances the physical and mechanical properties of cement-based materials. However, research on the modification mechanisms of nano-TiO_2_ on the mechanical properties, hydration process, and pore structure of concrete under low-temperature conditions remains relatively scarce, particularly lacking systematic experimental data at 5 °C, which is a typical winter construction temperature.

Based on the current research status and engineering requirements, this study simulates a low-temperature curing environment of 5 °C and focuses on the modification effect of nano-TiO_2_ on the early-age properties of concrete. In the experiments, nano-TiO_2_ was added to the concrete at three different dosages: 1%, 2%, and 3%. A control group of baseline concrete without nano-TiO_2_ addition was also included. Through a combination of mechanical property testing, differential thermal analysis (DTA), and micro-CT microporosity characterization, this study systematically investigates the following core issues: (1) The influence patterns of nano-TiO_2_ dosage on the compressive strength and splitting tensile strength of low-temperature concrete at 1d, 2d, 3d, 7d, and 28d ages, identifying the key factors controlling strength development. (2) Utilizing DTA to investigate the regulatory mechanism of nano-TiO_2_ on the formation of cement hydration products (particularly Ca(OH)_2_), revealing its acceleration pathway for low-temperature hydration processes. (3) CT scanning technology is a microscopic testing method that focuses on the micrometer-scale pore network structure, which dominates the mechanical properties of concrete. It specifically examines the distribution and morphological characteristics of harmful pores and interconnected pores within the internal structure. (4) Determine the optimal dosage of nano-TiO_2_ at 5 °C to provide experimental evidence and technical support for material design and engineering applications in winter concrete construction.

## 2. Materials and Mix Design

### 2.1. Raw Materials

**Figure 1:**
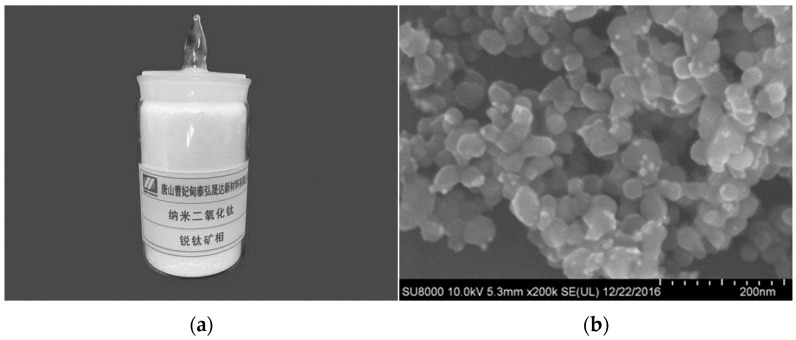
Nano-TiO_2_. (**a**) TiO_2_ sample. (**b**) SEM image.

The test employed P·O42.5 grade ordinary Portland cement, and its chemical composition and physical properties are shown in Table 1 and Table 2, respectively. The experiment utilized nano-TiO_2_ produced by Tangshan Caofeidian Taihong Shengda New Materials Co., Ltd., (Tangshan, China) which was incorporated into concrete as a cement replacement agent. Its physical properties are shown in Table 3. Sample images and SEM images are shown in Figure 1. To improve concrete workability, a polycarboxylic acid-based high-efficiency water-reducing agent (with a water-reduction rate of 34%) was used in the test. Concurrently, a low-alkali liquid accelerator for concrete was selected, with its technical properties listed in Table 4.

### 2.2. Specimen Preparation and Curing

**Figure 2:**
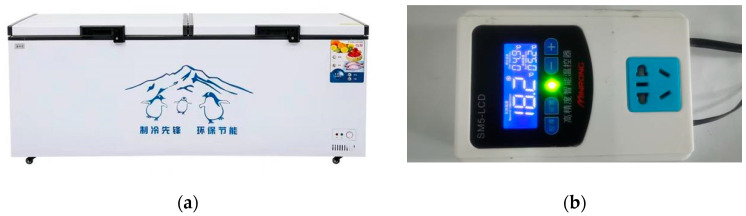
Low-temperature curing simulation apparatus. (**a**) Constant-temperature refrigerator. (**b**) Digital display controller.

To investigate the modification effects of different nano-TiO_2_ dosages on concrete under a 5 °C curing environment, a corresponding experimental plan was designed. We set the temperature to 5 °C using a digital temperature controller. The specific simulation apparatus is shown in Figure 2. Nano-TiO_2_ dosages of 1%, 2%, and 3% by cement mass were selected to investigate their effects on the mechanical properties of concrete.

In the experiment, sodium hexametaphosphate was used as a dispersant to improve the dispersion of nano-TiO_2_ particles. The specific parameters are shown in Table 5. The concrete mixing process is as follows: First, the dispersant is mixed with water. Then, the nanomaterials undergo 6 min of high-speed mechanical stirring to achieve dispersion. Finally, the mixture of nanomaterials and water is stirred together with aggregates and cement. Concrete without nano-TiO_2_ serves as the control group (JZ). The mix proportions for each group are shown in Table 6.

## 3. Experimental Methods

### 3.1. Basic Mechanical Properties Testing

During trial molding, nano-TiO_2_ was internally mixed into concrete based on the mass ratio of cementitious materials, with nano-type and nano-dosing serving as variables. By adjusting the dosage of water reducer, we controlled the slump of titanium dioxide-modified concrete within the range of 170 mm to 190 mm. After specimen molding was completed, the specimens were immediately placed in a low-temperature curing chamber for freeze–thaw curing. Mechanical property tests were conducted at 1-day, 2-day, 3-day, 7-day, and 28-day ages.

The specimen size for mechanical property testing is a 100 mm cube. For each curing age, seven working conditions are formed, with two sets of specimens (three specimens per set) produced for each condition. One set is used for compressive strength testing, and the other set is used for splitting tensile strength testing. The testing equipment used was an electro-hydraulic servo universal testing machine with a compressive load capacity of 1000 kN. The final result obtained was the average value of the measurements from three specimens. If the difference between any measured value and the median exceeds 15% of the median, the median shall be taken as the measured value. If the difference between two measured values and the median both exceed the specified limit, the test results for that group shall be deemed as invalid.

**Figure 3:**
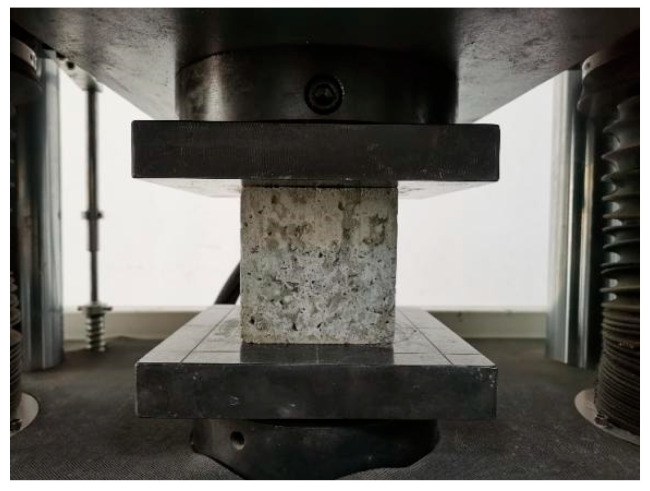
Compressive strength testing process.

Compressive strength testing shall be conducted in accordance with the Standard Test Methods for Physical and Mechanical Properties of Concrete (GB/T 50081-2019 [33]). Verify that the sides correspond to the upper and lower compression surfaces. During loading, the loading rate was set to 0.5 MPa/s. The specimen was observed until complete failure, and the ultimate failure load F was recorded. The test process is shown in Figure 3.

The compressive strength of concrete is determined according to Equation (1).

(1) $$f_{c}=\beta_{c}\frac{F_{c}}{A_{c}}$$

where $f_{c}$ is the compressive strength of concrete (MPa); $F_{c}$ is the compressive failure load of the specimen (kN); $A_{c}$ is the bearing area of the specimen (mm^2^); $\beta_{c}$ is the compressive strength reduction factor, taken as 0.95 for specimens with a side length of 100 mm.

The splitting tensile strength test was conducted in accordance with the Standard Test Methods for Physical and Mechanical Properties of Concrete (GB/T 50081-2019). As specified by the standard, the instrument was set to force control mode with a specimen loading rate of 0.05 MPa/s.

The splitting tensile strength of concrete is calculated according to Equation (2):

(2) $$f_{st}=\beta_{st}\frac{2F_{st}}{\pi A_{st}}=0.637\beta_{st}\frac{F_{st}}{A_{st}}$$

where $f_{st}$ is the splitting tensile strength of concrete (MPa); $F_{st}$ is the splitting tensile failure load of the specimen (N); $A_{st}$ is the splitting surface area of the specimen (mm^2^); $\beta_{st}$ is the splitting tensile strength reduction factor, taken as 0.95 for specimens with a side length of 100 mm.

### 3.2. Net Slurry Setting Time Test

Determine the initial setting time and final setting time of the pure mortar according to the method specified in the standard “Test Method for Strength of Cement Mortar (ISO Method)” (GB/T 17671-1999 [34]), with tests conducted at 20 s intervals.

The testing steps are as follows:

1. Weigh cement and water according to the specified mix ratio, then mix them into a uniform slurry using the designated mixing equipment.
2. Pour the pure slurry into the test mold, level it, and place it in a constant temperature and humidity curing chamber for curing.
3. After the specified time has elapsed, measure using the Vicat needle until the penetration depth meets the criteria for determining initial setting and final setting.

The testing equipment for pure paste setting time must meet the following requirements: Vicat needle specifications must comply with standards, with precise verticality and load accuracy. Mixing equipment must be compliant, and mixing parameters must conform to specifications. The curing chamber must maintain a temperature of 20 ± 1 °C and humidity ≥ 90%. Auxiliary equipment, including balances (accuracy ≤ 0.1g), thermometers (accuracy ≤ 0.5 °C), and test molds, must meet applicable standards.

### 3.3. Differential Thermal Analysis Testing

Differential thermal analysis identifies unknown substances by exploiting differences in the physical or chemical changes they undergo in response to temperature changes. Typically, experiments place an unknown substance alongside an equal amount of a known substance with stable chemical and physical properties under identical environmental conditions and variable temperature settings. The surface temperature difference between the two substances is then compared. By observing whether the temperature difference curve rises or falls, it is determined whether the substance undergoes an exothermic or endothermic reaction. The instrument used in the experiment was the ZCT Simultaneous Thermal Analyzer manufactured by Beijing Jingyi Gaoke Instrument Co., Ltd. (Beijing, China). Detailed specifications are provided in Table 7.

The specific testing procedure is as follows:

Remove the pure mortar specimens cured at low temperature to the specified age. Cut 5 mm cubes from the specimens using a small knife and immerse them in anhydrous ethanol for one day to terminate the hydration reaction. Prior to testing, dry the specimens in an oven at 80 °C for two hours. After drying, grind the specimens into powder. Then, using a specially designed small crucible, place 13.5 mg of powder on the right side of the machine. Place an empty crucible on the left side of the machine.

During testing, the instrument was set with an initial sampling temperature of 50 °C, a heating rate of 10 °C/min, a final temperature of 1000 °C, and a cooling rate of 5 °C/min.

### 3.4. Pore Structure Testing Method

Since the creation of micro-CT technology, its rapid development and expansion into the civil engineering materials industry have been driven by its non-destructive imaging capabilities, environmental safety, and other key features. Micro-CT captures perspective projections of samples from various angles using a flat-panel detector. These projections are processed by a computer system to generate high-resolution 3D images with resolution down to the pore scale, providing a novel approach for studying the pore structure of concrete. During CT imaging, X-rays emitted from the radiation source are attenuated by the object under examination. A detection plate converts the scanned information into electrical signals, which are then transmitted to a computer via digital-to-analog conversion. The computer generates a digital matrix based on the object’s X-ray absorption coefficient, analyzes the gray values, and arranges them into a CT image. Combined with three-dimensional reconstruction technology, this process enables micrometer-level digital three-dimensional characterization of the sample’s internal microstructure.

Pore structure testing utilizes the Saning Precision Instruments nanoVoxel-1000 desktop micro-CT system (Tianjin, China), which is equipped with VoxelStudio Scan software 2.5.1.25 for scanning and VoxelStudio Recon software for 3D reconstruction. The former enables parameter configuration based on sample type, controls device positioning, and completes scanning to acquire raw data for reconstruction. The latter reconstructs scanned data into 3D volumetric data for visualization and analysis.

**Figure 4:**
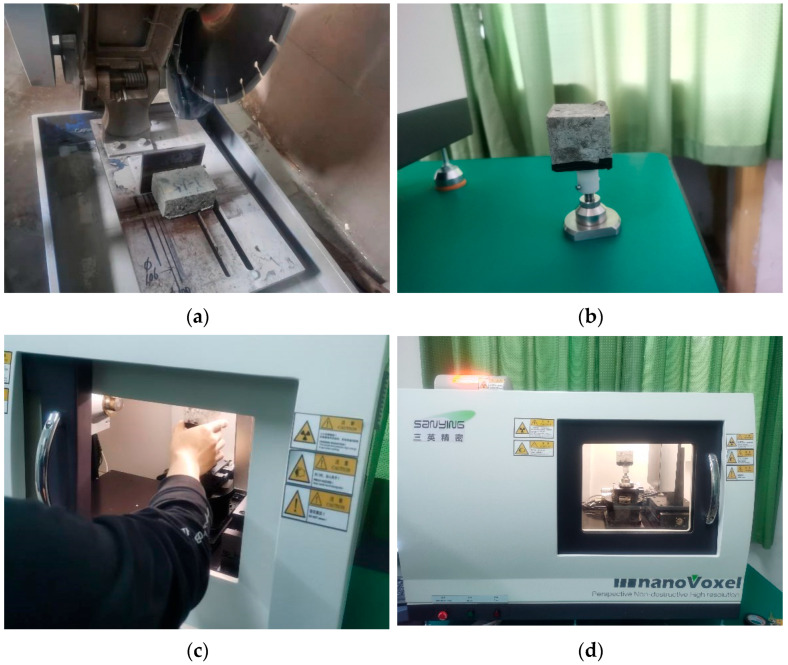
Experimental procedure. (**a**) Cut specimens. (**b**) Place the core sample on the stage. (**c**) Place the sample into the CT scanner. (**d**) Perform a scan.

Before scanning, form a 100 mm × 100 mm × 100 mm cubic concrete block and place it in a constant-temperature curing chamber. Remove the block after reaching the specified curing age. Then cut the test block into 50 mm × 50 mm × 50 mm small cubic specimens, mark them, and place them on the sample stage. Finally, place the specimens along with the stage into the scanning equipment for analysis. Refer to Figure 4a–d for specific steps.

After scanning to obtain the sample’s 3D volumetric data, subsequent processing was performed using Dragonfly from Canada’s ORS and the digital core analysis software Sypicore: Dragonfly enables refined 3D visualization characterization such as image cropping, denoising, and segmentation, while Sypicore excels at pore–fracture binary segmentation and quantitative analysis of pore network topology.

Due to variations in material density, CT slice images exhibit differing grayscale levels (low grayscale represents voids and cracks, medium grayscale indicates cement matrix, and high grayscale corresponds to aggregates). Issues such as blurred boundaries, systematic artifacts, and information loss caused by improper parameter adjustments necessitate image processing using Dragonfly prior to void extraction. Specific workflow (Figure 4): Import 3D volumetric data to construct and render images, then crop to remove irrelevant sections. Apply filtering for noise reduction and threshold segmentation to define regions of interest (ROIs), finally extracting and analyzing voids and cracks.

## 4. Experimental Results

### 4.1. Basic Mechanical Properties

**Figure 5:**
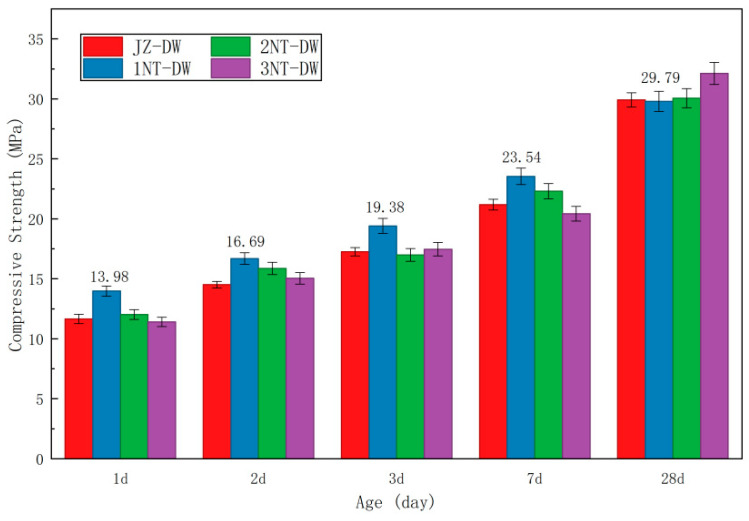
Compressive strength of concrete at different TiO_2_ nano-doping levels.

**Figure 6:**
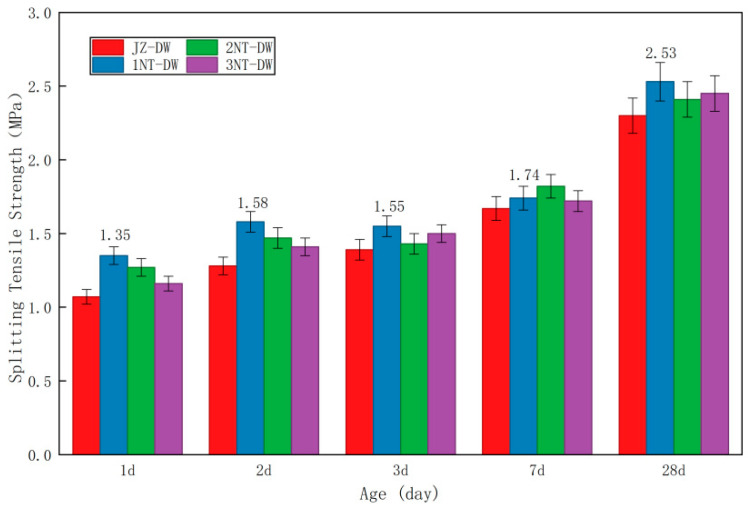
Splitting tensile strength of concrete at different TiO_2_ nano-doping levels.

Table 8 presents the compressive strength and splitting tensile strength values of concrete cubes with different nano-TiO_2_ dosages obtained from experiments. The compressive strength values are reduced by a factor of 0.95, while the splitting tensile strength values are reduced by a factor of 0.85. Figure 5 and Figure 6 present the compressive strength and splitting tensile strength of concrete cubes at different nano-TiO_2_ dosages.

As shown in the figure above, at a low temperature of 5 °C, the strength development patterns of concrete exhibit consistent trends across different admixture dosages. Specifically, concrete strength increases rapidly during the early stages, with the strength at 1 day reaching nearly half of the strength at 28 days. At the 1-day age, the compressive strength under standard conditions had reached 39% of the 28-day strength, while the tensile splitting strength had reached 47% of the 28-day strength. Regarding compressive strength, the development patterns of nano-TiO_2_ at different dosages at 1 d, 2 d, 3 d, and 7 d exhibited a “peak-shaped” curve. Specifically, the JZ-DW condition showed the lowest strength, which increased sharply after adding 1% nano-TiO_2_, and then the strength improvement effect began to decline as the dosage increased. After the 28-day age, the strength differences among various admixture dosages decreased and generally converged. For splitting tensile strength, at the 1-day, 2-day, and 3-day ages, the 1NT-DW dosage consistently exhibited the highest strength among all admixture conditions. Compared to the control condition, the 1NT-DW dosage showed increases of 25%, 23%, and 12%, respectively. By the 7-day and 28-day ages, the splitting tensile strengths began to converge.

In summary, the addition of nano-TiO_2_ enhances concrete strength in both compressive strength and splitting tensile strength compared to the baseline condition. This improvement is particularly pronounced during the early-age stage, with the strengthening effect gradually diminishing after 28 days. Among all dosage scenarios, a 1% nano-TiO_2_ content yields the most significant enhancement in concrete mechanical properties under low-temperature conditions.

### 4.2. Net Slurry Setting Test

**Figure 7:**
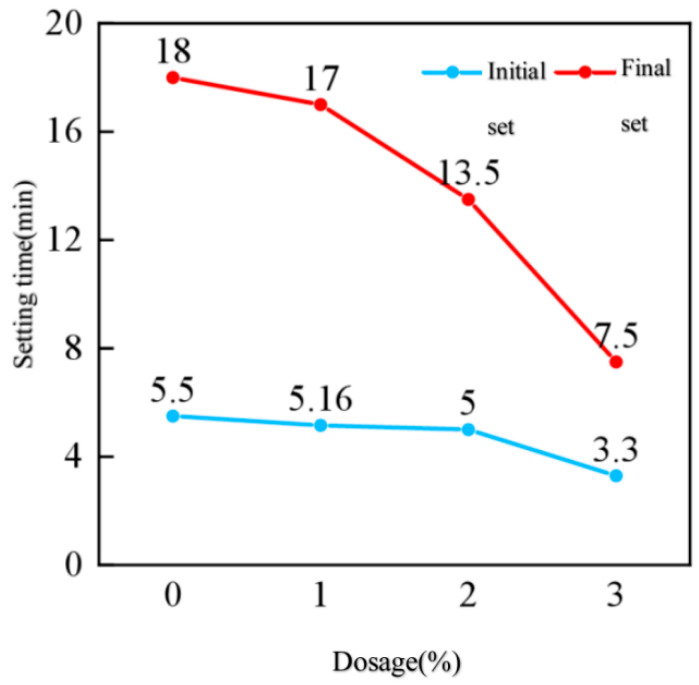
Setting time of net slurry under different TiO_2_ nano-dispersions.

Figure 7 shows the setting times of cement paste under different nano-TiO_2_ dosages. The figure indicates that the nanomaterial accelerates both the initial and final setting of cement paste. Under the influence of nano-TiO_2_, both the initial and final setting times of cement paste decrease progressively with increasing dosage. Without nano-TiO_2_ addition, the initial setting time and final setting time of the cement paste were 5.5 min and 18 min, respectively. When nano-TiO_2_ was added, the effect of nano-TiO_2_ dosage on setting time during the cement paste’s initial setting process occurred in two distinct phases: at dosages of 1% and 2%, the impact on setting time was relatively minor. At a dosage of 3%, the effect on initial setting time suddenly increased significantly, drastically shortening the cement paste setting time, with the initial setting time rapidly decreasing to 3.3 min. During the final setting of the cement paste, the setting time continuously decreased with increasing dosage.

### 4.3. Differential Thermal Analysis

**Figure 8:**
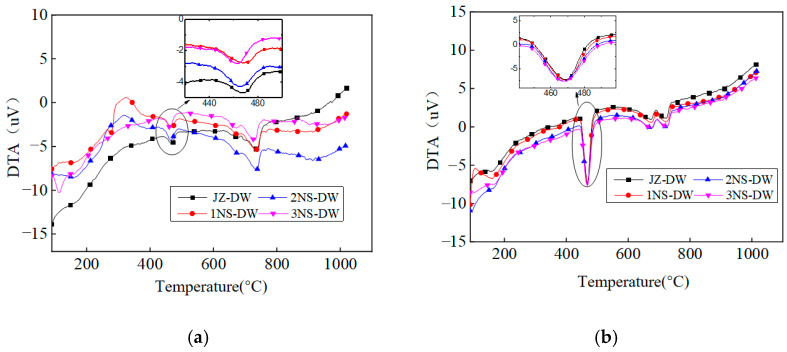
DTA curves of pure slurry at different TiO_2_ nano-doping levels. (**a**) 1d Pure Pulp DTA curve. (**b**) 7d Pure Pulp DTA curve.

As shown in Figure 8, the DTA curves for each nano-TiO_2_ loading also exhibits two distinct endothermic peaks. Figure 8a and Figure 8b represent the curves for 1-day and 7-day aged samples, respectively. The figures indicate that the area of the Ca(OH)_2_ endothermic peak gradually increases with aging duration.

**Figure 9:**
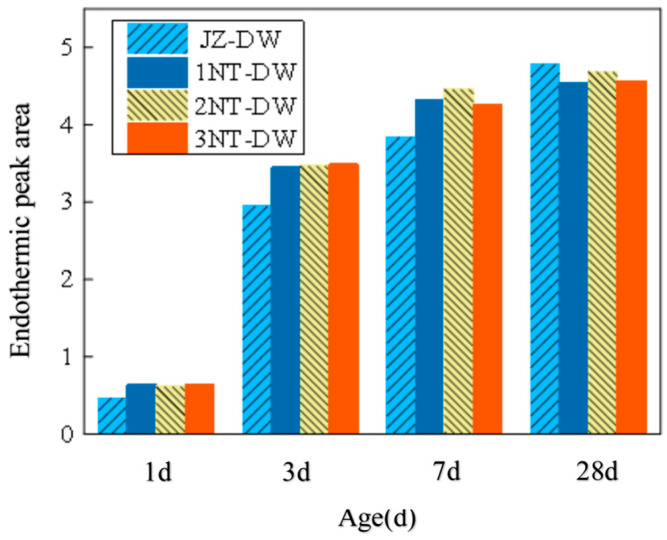
Area of the endothermic peak of Ca(OH)_2_ at different ages.

Figure 9 shows the heat absorption peak areas of Ca(OH)_2_ at different ages under the influence of nano-TiO_2_ addition. As seen in the figure, the heat absorption peak area of Ca(OH)_2_ continuously increases with age under low-temperature conditions. The expansion rate is relatively rapid before the 3-day age, gradually slowing down thereafter.

At the 1 d aging stage, the figure shows that the heat absorption peak area of Ca(OH)_2_ for the nano-TiO_2_ addition condition is larger than that for the non-addition condition. Moreover, the heat absorption peak areas for TiO_2_ addition levels of 1%, 2%, and 3% are consistent. Compared to the unmodified condition, the heat absorption peak area increased by 36% with 1% TiO_2_ content, by 32% with 2% TiO_2_ content, and by 39% with 3% TiO_2_ content. At the 3-day age, the heat absorption peak area of Ca(OH)_2_ under nano-TiO_2_ addition conditions remained larger than that under non-addition conditions. The heat absorption peak areas for 1%, 2%, and 3% addition levels increased by 17%, 18%, and 18%, respectively, compared to the non-addition condition. By the 7-day age, the increases in heat absorption peak area for 1%, 2%, and 3% additions decreased to 12%, 16%, and 11%, respectively. At the 28-day age, the heat absorption peak area of Ca(OH)_2_ with nanomaterial additions was actually smaller than that of the non-added control.

In summary, the incorporation of nano-TiO_2_ further accelerates the early-stage hydration reaction of cement, with the effect being more pronounced at the 1-day age. As the dosage gradually increases, the Ca(OH)_2_ content in the hydration products remains essentially unchanged, indicating that beyond a 1% dosage, further increases have a negligible impact on enhancing the cement hydration reaction. It can be seen that the pattern of promoting cement hydration by the dosage does not align with the strength pattern. The primary reason lies in the fact that in concrete, a higher dosage leads to an excess of nano-TiO_2_, and this excess nano-TiO_2_ forms small agglomerates, thereby exerting a negative effect on strength. However, for cement paste, where higher dosages are used, the small agglomerates formed by nano-TiO_2_ do not affect the relevant hydration rates. Therefore, beyond a dosage of 1%, the rate of hydration changes less rapidly as the dosage increases.

### 4.4. Microscopic Pore Structure Characteristics

**Figure 10:**
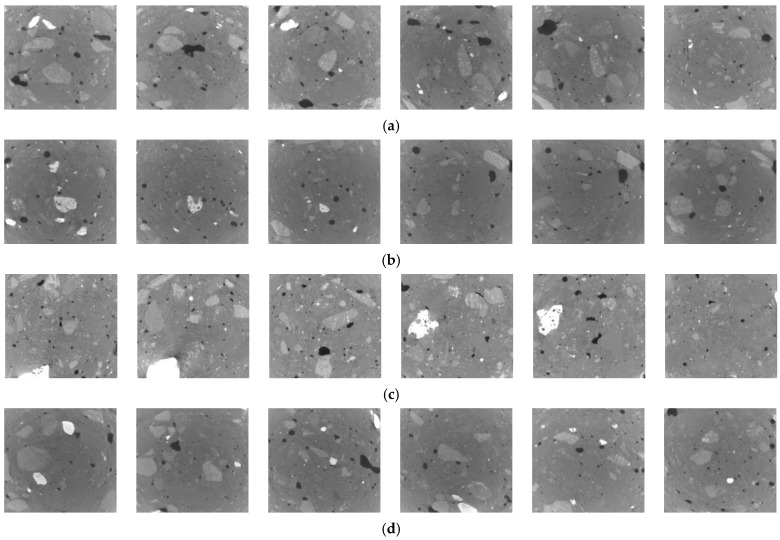
Cross-sectional view of pore structure in concrete with different TiO_2_ nano-dispersions at 1 d age. (**a**) Cross-sectional diagram of pore structure changes at age 1 d under JZ-DW operating conditions. (**b**) Cross-sectional view of pore structure changes at 1 d age under 1NT-DW conditions. (**c**) Cross-sectional view of pore structure changes at 1 day age under 2NT-DW operating conditions. (**d**) Cross-sectional view of pore structure changes at 1 day age under 3NT-DW operating conditions.

Figure 10 shows cross-sectional images of the pore structure in concrete at the 1-day age with different nano-TiO_2_ dosages. Each set of images displays 100, 200, 300, 400, 500, and 600 cross-sectional images from left to right. Comparing the JZ-DW, 1NT-DW, 2NT-DW, and 3NT-DW conditions reveals that after modification with nano-TiO_2_, the pore distribution in the concrete becomes more regular, and the number of pores decreases. Cross-sectional views of the pore structures at different nano-TiO_2_ loading levels show minimal variation, with no significant distinction in pore count or pore size. This indicates that incorporating nano-TiO_2_ effectively optimizes pore distribution and reduces pore density. However, distinguishing the pore structures at different nano-TiO_2_ loadings through cross-sectional analysis alone is challenging and requires further investigation.

To further characterize the pore structure parameters of concrete at the microscopic scale under various admixture dosages, this study employs Sypi Core digital core software to analyze the pore network model of the 3D volume constructed from CT scans. Internal pore features are extracted as similar topological structures using the software’s built-in maximum sphere algorithm, enabling quantitative analysis of pore structure parameters.

The core of the pore network model lies in representing geometrically complex pore structures as equivalent spatial configurations composed of simple geometric bodies. The primary algorithm employed in the software is the maximum sphere algorithm, a computational method applied to pore network modeling. This approach generally encompasses two concepts: maximum spheres and maximum sphere clusters. The construction of the maximum ball network model primarily involves two steps: first, establishing the inscribed spheres; second, removing redundant spheres. The mesh within the 3D digital rock core is termed voxels, representing the smallest volumetric units within the three-dimensional rock core. Starting from the pore voxels within the digital rock core and extending outward toward the skeletal voxels, the collective set of these extended bodies is referred to as the inscribed spheres. If the largest inscribed sphere exists within the same region, then all sub-inscribed spheres contained within this inscribed sphere are termed redundant spheres, and this largest inscribed sphere is termed the maximum sphere. Ultimately, the set of maximum spheres can characterize the entire pore space without redundancy. Subsequently, based on the radius and rank of the maximum spheres, a tree structure and clustering algorithm are employed to determine the pores and throats within the pore structure. Finally, the pore structure is segmented, and the dimensions of the pores and throats are calculated.

**Figure 11:**
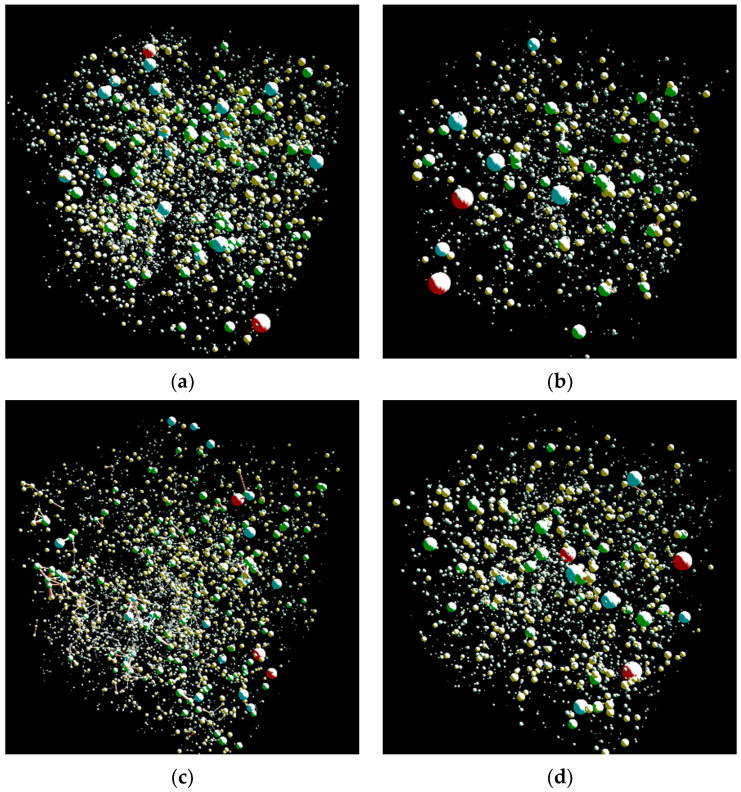
Pore network model of Nano-TiO_2_ at different dosages at the 1 d aging stage. (**a**) JZ-DW. (**b**) 1NT-DW. (**c**) 2NT-DW. (**d**) 3NT-DW.

Figure 11 presents the three-dimensional pore network models for various TiO_2_ nano-doses at the 1-day age. The image clearly shows that the JZ-DW concrete matrix is filled with pore spheres of varying sizes. Smaller pore spheres exhibit dense and widespread distribution, while larger pore spheres predominantly cluster around the cube centers. Comparing the pore network models of conditions 1NT-DW, 2NT-DW, and 3NT-DW with JZ-DW reveals that nano-TiO_2_ addition also reduces the number and density of pore spheres within the concrete. Among the three dosages, condition 1NT-DW exhibits the fewest small pore spheres and the lowest overall pore sphere distribution density. The 2NT-DW and 3NT-DW conditions exhibit higher numbers of pore spheres and greater distribution densities. In summary, based on the ranking of effectiveness in influencing pore structure distribution, the 1% nano-TiO_2_ dosage yields the optimal results among the different dosages tested.

Table 9 presents the calculated micro-pore parameters of the pore network model at various admixture dosages. As shown in the table, the micro-pore volume fraction of JZ-DW concrete at 1 day was 2.74%. After nanomaterial addition, the micro-pore volume fraction decreased significantly: 1NT-DW, 2NT-DW, and 3NT-DW concrete exhibited micro-pore volume fractions of 1.50%, 2.22%, and 1.98%, respectively. with 1NT-DW exhibiting the lowest micro-pore volume fraction, followed by 3NT-DW. Compared to JZ-DW, the pore volumes decreased by 45%, 19%, and 28%, respectively. This demonstrates that under low-temperature conditions, the unique “nano-effect” of nanomaterials significantly optimizes the pore structure of concrete during the early age. Similar patterns can be observed by analyzing other parameters in the pore network model.

In summary, the incorporation of nanomaterials significantly improves the pore structure of concrete at the 1-day age. Among the various TiO_2_ nano-dispersions tested, the 1% dosage yielded the most favorable results in terms of pore structure enhancement.

**Figure 12:**
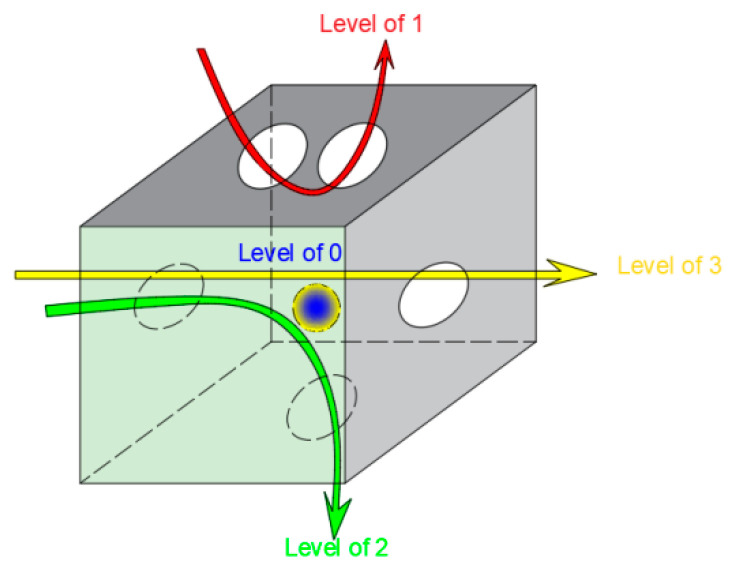
Schematic diagram of pore connectivity classification.

To further characterize the connectivity of micro-pores in concrete at different admixture dosages, this study performs a connected pore domain analysis on the three-dimensional concrete volume after threshold segmentation. First, connected pores are classified into four categories (Figure 12: Schematic Diagram of Connected Pore Classification): Level 1 connected pores, representing pores connected to one surface of a cube; Level 2 connected pores, representing pores connected to two adjacent surfaces of a cube; and Level 3 connected pores, representing pores connected from one surface to the opposite surface. The connected domain analysis software employed in this study is Connected Pores, which enables quantitative evaluation of the number of pores, equivalent diameter, and connectivity level within the three-dimensional volume.

**Figure 13:**
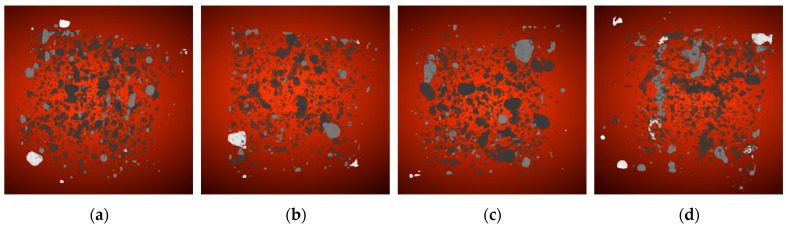
Analysis of connected domains in concrete at 1 day age with different TiO_2_ nano-additive dosages. (**a**) JZ-DW. (**b**) 1NT-DW. (**c**) 2NT-DW. (**d**) 3NT-DW.

Figure 13 shows the connectivity domain analysis of nano-TiO_2_ at different dosages at the 1-day age. To facilitate distinguishing connectivity domains of different levels, connectivity pores of levels 0, 1, 2, and 3 are labeled as black, gray, white, and brown, respectively. The figure reveals that compared to concrete without nanomaterial addition, the incorporation of nanomaterials significantly alters the internal connectivity domain structure. The distribution of connected pores becomes more dispersed, with a marked reduction in all connectivity levels, particularly in zero-level and first-level connected pores.

Table 10 presents the connectivity porosity levels for different TiO_2_ nano-dispersions after connectivity domain analysis. The table indicates that Level 0 connectivity porosity accounts for the highest proportion across all connectivity pores. Simultaneously, the porosity decreases to varying degrees with increasing nanomaterial content. Under JZ-DW conditions, Level 0 connectivity porosity is 2%, constituting 72.9% of the total connectivity porosity across all four categories. For the 1NT-DW, 2NT-DW, and 3NT-DW conditions, the zero-level connected porosity was 1.11%, 1.59%, and 1.44%, respectively, representing reductions of 44%, 21%, and 28% compared to the JZ-DW condition. Level 1 interconnected pores constitute the second-largest proportion of total interconnected pores after Level 0. Their porosity variation pattern with nanomaterial addition aligns with that of Level 0 interconnected pores. Level 2 interconnected pores exhibit an extremely low proportion compared to Levels 0 and 1. Their porosity remains relatively stable at approximately 1.0% across all nanomaterial dosages. Level 3 interconnected pores exhibited 0% porosity across all nanomaterial dosages, indicating the absence of pore networks connecting opposing surfaces of the cube within the concrete at the early 1-day age.

In summary, Level 0 connected pores constitute a significant proportion in the microporous structure of concrete, while Levels 1, 2, and 3 connected pores account for a smaller proportion. With the incorporation of nanomaterials, the connectivity of the concrete microstructure decreases. Among different TiO_2_ nano-filler dosages, the 1% dosage exhibits the lowest connectivity. The addition of NT directly affects the porosity of zero-level and first-level connected pores, while having a minor impact on second-level and third-level connected pores. This indicates that nanomaterials primarily affect the numerous isolated zero-level interconnected pores within the concrete and some surface-connected Level 1 interconnected pores, thereby reducing the overall connectivity of the concrete. As the dosage increases, the connectivity of concrete pores decreases. One reason for this is that nanomaterials require an appropriate dosage, which is highly effective for uniform particle dispersion and pore filling.

## 5. Discussion

Based on experimental data and materials science theory, the modification effect of nano-TiO_2_ on low-temperature concrete is primarily achieved through the synergistic action of nucleation effect, filling effect, and hydration regulation effect. The contribution of these three effects varies significantly across different curing ages, as detailed below:

1. Nucleation Effect: As a key driver for early strength development, low-temperature environments significantly retard cement hydration, resulting in slow and uneven formation of hydration products (C-S-H gel, Ca(OH)_2_ and calcium aluminate hydrate AFt). This is the primary cause of low early strength in concrete. Nano-TiO_2_ particles (particle size 20 ± 5 nm, specific surface area 85 m^2^/g) possess extremely high surface energy, acting as nucleation sites for heterogeneous precipitation of hydration products. This accelerates the crystallization growth of C-S-H gel and Ca(OH)_2_. Differential thermal analysis (DTA) results indicate that at the 1-day age, the heat absorption peak area of Ca(OH)_2_ in all nano-TiO_2_ blended groups increased by 32–39% compared to the control group (JZ-DW). Furthermore, no significant difference in peak area was observed among the 1%, 2%, and 3% blending groups, demonstrating that the nucleation effect of nano-TiO_2_ in the early stage is unaffected by dosage (within the 1–3% range). Only a small number of particles are required to provide sufficient nucleation sites for hydration products. 3% TiO_2_ groups showed no significant difference in peak area, indicating that the nucleation effect of nano-TiO_2_ in the early stage is unaffected by dosage (within the 1–3% range). Only a small number of particles are required to provide sufficient growth sites for hydration products.

Mechanical property data further corroborates this mechanism: At the 1-day age, the compressive strength of the 1% dosage group (1NT-DW) reached 13.98 MPa, representing a 20% increase compared to the control group (11.65 MPa). while the splitting tensile strength reached 1.35 MPa, a 26% increase over the control group (1.07 MPa). The strength enhancement at this dosage remained the highest at 2 and 3 days of age (18% increase in compressive strength at 2 days and 12% at 3 days). This indicates that the nucleation effect dominates during the early age (1–3 days), directly shortening the “induction period” of cement hydration and enabling concrete to rapidly form a strength skeleton even at low temperatures.

2. Filling Effect: The core pathway for optimizing micro-pore structure. The macroscopic properties of concrete are directly correlated with its micro-pore characteristics. Low-temperature curing readily leads to the formation of numerous interconnected pores and large-aperture defects within the concrete matrix, reducing structural compactness. Nano-TiO_2_ particles, which are significantly smaller than cement particles (typically 1–100 μm in size), refine the pore structure through a “gradient filling” mechanism: they fill the primary pores between cement particles while simultaneously blocking secondary pores formed during hydration product development, thereby reducing pore connectivity. Based on micro-CT scanning and pore network modeling analysis, at the 1-day age, the porosity of the 1NT-DW group was only 1.50%, which is a 45% reduction compared to the control group (2.74%). The number of pores decreased from 3308 to 1850, and the average pore volume decreased from 3.311 × 10^8^ μm^3^ to 2.614 × 10^8^ μm^3^. Simultaneously, the connectivity level 0 porosity (isolated pores) decreased from 2.00% to 1.11%, and the connectivity Level 1 porosity (single-surface connected pores) decreased from 0.62% to 0.35%. Meanwhile, the connectivity Levels 2 and 3 porosities remained at extremely low levels (<0.14%). This result indicates that the filling effect of nano-TiO_2_ can significantly reduce both the “number” and “connectivity” of pores within concrete, demonstrating particularly pronounced optimization effects on isolated pores and surface-connected pores that readily form during the early stages. The 1% dosage group exhibited the most optimized pore structure, further demonstrating that an appropriate amount of nano-TiO_2_ achieves “precision filling”. Excessive amounts, however, may lead to reduced filling efficiency due to particle agglomeration (the 3NT-DW group had a porosity of 1.98%, which is higher than the 1NT-DW group).

3. Hydration Regulation Effect: The equilibrium mechanism between setting time and hydration rate. Extended setting times in concrete at low temperatures increase construction difficulty. Nano-TiO_2_ can reasonably shorten setting time by regulating cement hydration rate. According to pure paste setting tests, without nano-TiO_2_ addition, initial setting time was 5.5 min and final setting time was 18 min, and with 3% nano-TiO_2_ addition, initial setting time sharply decreased to 3.3 min and final setting time shortened to 12 min. Setting time exhibited a “stepwise decrease” with increasing dosage: 1% and 2% additions had minor effects (initial setting around 5.0 min), while 3% addition caused a sudden acceleration.

The essence of this phenomenon lies in the regulation of cement hydration pathways by nano-TiO_2_: at low dosages (1–2%), nanoparticles primarily promote hydration product formation through nucleation effects, exerting a relatively moderate overall influence on hydration rates. At high dosages (3%), excess particles adsorb moisture from cement particle surfaces, increasing local ion concentrations (Ca^2+^, SiO_4_^4−^) and accelerating the reaction between C_3_A and gypsum (forming AFt), thereby significantly shortening setting time. However, it should be noted that although setting speed increases at a 3% dosage, the 1-day compressive strength (11.41 MPa) is lower than that of the control group. This indicates that excessive acceleration of setting may lead to uneven distribution of hydration products, resulting in localized structural defects.

## 6. Conclusions

This study employed an indoor experimental setup to simulate the low-temperature environment at tunnel portals after thermal insulation measures were implemented. It investigated the effects of various nano-modified dosages on concrete’s mechanical properties, hydration reaction characteristics, and micro-pore structure, while also determining the optimal dosages for two types of nanomaterials. The primary conclusions are as follows:

(1) In low-temperature environments, the addition of nanomaterials can significantly enhance the early-age strength of concrete. At the 1-day age, concrete containing 2% and 1% nano-TiO_2_ exhibits compressive strength and splitting tensile strength exceeding 40% of the 28-day strength.
(2) The enhancement effect on the early-stage mechanical properties of concrete under different nano-TiO_2_ dosages exhibits a “peak-shaped” curve, where the strength improvement first increases and then decreases with dosage. The optimal dosage of nano-TiO_2_ for achieving the best mechanical properties is 1%.
(3) After incorporating nanomaterials, the area of the Ca(OH)_2_ endothermic peak in 1-day-old pure mortar specimens increased significantly, Ca(OH)_2_ content rose markedly, and the hydration rate accelerated substantially.
(4) Parameters from the microscopic pore network model based on CT scanning technology indicate that nanomaterials can optimize the microscopic pore structure of concrete and reduce its connectivity. Among all dosage conditions, 1% nano-TiO_2_ showed the most significant improvement, which is consistent with the patterns observed in its mechanical properties.

## Figures and Tables

**Table 1:** Chemical composition of cement.

Chemical Composition	CaO	SiO_2_	Fe_2_O_3_	Al_2_O_3_	SO_3_	MgO	Na_2_O	C_3_A	C_3_S	C_2_S	C_4_AF
Content/%	64.5	21.7	3.4	4.6	0.4	3.5	0.6	6.3	56.6	19.8	10.4

**Table 2:** Physical properties of cement.

Cement Grade	Specific Surface Area (m^2^/kg)	Initial Setting Time (min)	Final Setting Time (min)
42.5	349	151	210

**Table 3:** Physical properties of nano-TiO_2_.

Model Specifications	TiO_2_ Content	Specific Surface Area (m^2^/g)	Particle Size (nm)	Bulk Density (g/L)
RT150	≥99.8%	85	20 ± 5	<500

**Table 4:** Technical properties of low-alkali liquid accelerator.

Total Alkalinity	Initial Setting Time	Final Setting Time	1d Compressive Strength	28-Day Strength Retention Rate
3.5%	2 min 30 s	7 min 50 s	8.0 MPa	87%

**Table 5:** Sodium hexametaphosphate.

Molecular Formula	Density (g/mL)	Melting Point (°C)	Refractive Index	Complexity	Molecular Weight
(NaPO_3_)_6_	2.5	616	1.482	513	611.17

**Table 6:** Mix proportions for concrete with different titanium dioxide nanoparticle dosages (unit: kg/m^3^).

Mix Design Number	Cement	Nano Titanium Dioxide	Sand	Crushed Stone	Water
JZ-DW	420.0	0.0	867	769	201
1NT-DW	415.8	4.2	867	769	201
2NT-DW	411.6	8.4	867	769	201
3NT-DW	407.6	12.6	867	769	201

**Table 7:** Parameters of the simultaneous thermal analyzer.

Model	Temperature Range	Heating Rate	Cooling Rate	Temperature Sensitivity	Differential Temperature Range
ZCT-1	Room temperature-1000 °C	0.1~100 °C/min	0.1~40 °C/min	0.1 °C	10~1000 µV

**Table 8:** Compressive strength and splitting tensile strength of concrete at different TiO_2_ nano-doping levels.

	Specimen Number	Nano-Level Dosage	1d	2d	3d	7d	28d
Compressive strength (MPa)	JZ-DW	/	11.65	14.18	17.26	21.18	29.91
	1NT-DW	1%	13.98	16.69	19.38	23.54	29.79
	2NT-DW	2%	12.02	15.87	16.99	22.31	30.06
	3NT-DW	3%	11.41	15.04	17.47	20.43	32.13
Splitting Tensile Strength (MPa)	JZ-DW	/	1.07	1.28	1.39	1.67	2.30
	1NT-DW	1%	1.35	1.58	1.55	1.74	2.53
	2NT-DW	2%	1.27	1.47	1.43	1.82	2.41
	3NT-DW	3%	1.16	1.41	1.50	1.72	2.45

**Table 9:** Microscopic pore parameters of pore network model.

Pore Structure Parameters		JZ-DW	1NT-DW	2NT-DW	3NT-DW
Porosity		2.74%	1.50%	2.22%	1.98%
Pore count		3308	1850	2457	2785
Pore volume (µm^3^)	Maximum value	1.906 × 10^11^	2.005 × 10^11^	1.648 × 10^11^	1.892 × 10^11^
	Average	3.311 × 10^8^	2.614 × 10^8^	2.168 × 10^8^	2.882 × 10^8^
Number of vocal tracts		418	237	284	249
Roar Volume (µm^3^)	Maximum value	5.850 × 10^10^	5.726 × 10^10^	5.498 × 10^10^	5.709 × 10^10^
	Average	6.916 × 10^7^	3.174 × 10^7^	4.127 × 10^7^	3.172 × 10^7^
Maximum coordination number		4	6	7	7

**Table 10:** Connectivity porosity at different levels for concrete at 1-day age.

Blending Conditions	Level 0 Connected Porosity (%)	Level 1 Connected Porosity (%)	Level 2 Connected Porosity (%)	Level 3 Connected Porosity (%)
JZ-DW	2.00	0.62	0.12	0
1NT-DW	1.11	0.35	0.04	0
2NT-DW	1.59	0.52	0.11	0
3NT-DW	1.44	0.40	0.14	0

## Data Availability

Data will be made available on request.
